# The relevance of a right scale for sampling when studying high-resolution behavioral dynamics

**DOI:** 10.1038/s41598-023-39295-z

**Published:** 2023-08-16

**Authors:** L. Barberis, C. Simian, R. H. Marin, J. M. Kembro

**Affiliations:** 1 Facultad de Matemática, Astronomía Física y Computación, Universidad Nacional de Córdoba, Córdoba, Argentina; 2 Instituto de Física Enrique Gaviola (IFEG), Consejo Nacional de Investigaciones Científicas y Técnicas (CONICET), Córdoba, Córdoba Argentina; 3 Instituto de Investigaciones Biológicas y Tecnológicas (IIByT), Consejo Nacional de Investigaciones Científicas y Técnicas (CONICET) - Universidad Nacional de Córdoba, Córdoba, Córdoba Argentina; 4 Instituto de Ciencia y Tecnología de los Alimentos (ICTA) and Cátedra de Química Biológica, Departamento de Química, Facultad de Ciencias Exactas, Físicas y Naturales, Universidad Nacional de Córdoba, Córdoba, Córdoba Argentina

**Keywords:** Animal behaviour, Complexity, Dynamical systems

## Abstract

Many species used in behavioral studies are small vertebrates with high metabolic rates and potentially enhanced temporal resolution of perception. Nevertheless, the selection of an appropriate scales to evaluate behavioral dynamics has received little attention. Herein, we studied the temporal organization of behaviors at fine-grain (i.e. sampling interval ≤1s) to gain insight into dynamics and to rethink how behavioral events are defined. We statistically explored high-resolution Japanese quail (*Coturnix japonica*) datasets encompassing 17 defined behaviors. We show that for the majority of these behaviors, events last predominately <300ms and can be shorter than 70ms. Insufficient sampling resolution, even in the order of 1s, of behaviors that involve spatial displacement (e.g. walking) yields distorted probability distributions of event durations and overestimation of event durations. Contrarily, behaviors without spatial displacement (e.g. vigilance) maintain non-Gaussian, power-law-type distributions indicative of long-term memory, independently of the sampling resolution evaluated. Since data probability distributions reflect underlying biological processes, our results highlight the importance of quantification of behavioral dynamics based on the temporal scale pertinent to the species, and data distribution. We propose a hierarchical model that links diverse types of behavioral definitions and distributions, and paves the way towards a statistical framework for defining behaviors.

## Introduction

Considering the West Brown Enquist law^1^, smaller animals have a faster metabolism, which in turn favors the possibility of performing faster body movements. Mice, *Mus musculus*, have been shown to move their whiskers at a speed of 25 Hz (1/25 s)^2^, and hens, *Gallus gallus domesticus*, can present rapid head movements associated with preening at 5 Hz (1/5 s)^3^. Interestingly, body size and metabolic rate have also been proposed to be linked with the perception of temporal information^4^, with fast movements of small animals being perceived by conspecifics as social cues^5^. Although, high-resolution data can hold biologically relevant information, especially within social interactions, the temporal organization of behaviors at resolution ≤1s has received little attention. This raises the question of whether a species-specific appropriate temporal scale is in general being used in behavioral studies. Scale in this context refers to both the grain of observation, given by the temporal resolution available within a given data set (considered “fine” at high and “coarse” at low resolutions respectively) and the extent (i.e. duration) of analysis^6^. Following the conceptualization of the “Umwelt”, the grain of the study should be in accord with the animal’s perception of its environment through its senses^7^.

Since animal behavior is not random, the relative frequency and duration of behaviors can only be approximated through sampling^8^. However, exactly how this should be achieved is a long withstanding debate^8,9^. In small animals, an apparently simple question, such as the total time spent performing a behavior or the duration of each behavioral event (i.e. continuous time spent performing a given behavior), could have strikingly different answers depending on the methodology implemented (reviewed in^8,9^). Behaviors can be defined associated with topography e.g. overall body movements and/or function^10^. When events are recorded at real time, some behaviors happen so rapidly that they can only be considered as an occurrence and not as a duration, while other can be considered as a state with longer meaningful durations^8^. However, if sufficient high-resolution data is analyzed, even behavioral states such as walking can appear more discontinuous than those visually observed, with pauses between steps becoming evident^11–13^. Thus the sampling interval (i.e. time between sample points) needed in order to be able to perform comprehensive analyses of behavioral dynamics is most likely associated with the type of behavior, as well as the species under study.

With the rise of computers in the 1970s, different approaches began to be used to gain understanding regarding the temporal organization of behavior based on the frequency and/or probability distribution of the duration of behavioral events and inter-events (i.e. pauses between events)^14^. On one hand, researchers recognized that a behavior is usually clustered in bouts^15^. On the other hand, within the framework of complex systems and scaling, studies show that the duration of inter-event periods associated with movement can be described as a power law (i.e. a straight line in a double logarithmic plot) in a wide variety of species, for example, flies *Drosophila melanogaster*^16^, humans *Homo sapiens*^17^, rats *Rattus norvegicus*^18^ and Japanese quail *Coturnix japonica*^11–13^. Power laws indicate that the underlying dynamic mechanism have self-similar statistical properties on different time scales^17^. This statistical self-similarity is a defining characteristic of fractal objects. Hence, the presence of a power law distribution has strong theoretical implications given that it implies that behavioral time series can be considered an output of a complex dynamical system, presenting long-range (auto) correlations associated with processes with long-term memory^19^.

The heated discussion associated with temporal organization, bouts, scaling, and complexity, in general, has not yet percolated to the rest of the scientific community. Most research continues to implicitly assume the absence of auto-correlations^20^ between data points within behavioral time series, and a Gaussian distribution. This is evidenced by the fact that the most frequently used variables in behavioral studies imply averaging temporal data to extract a mean value, without checking if such a quantity has meaning as a representative time. In this regard, few studies have actually analyzed the probability distribution of duration of different types of behavioral events and, even less, how distributions change in different experimental contexts.

The aim of this study is to gain insight into the temporal dynamics of behavior by rethinking the definition of behavioral events. Using datasets of behavioral time series of Japanese quail (*Coturnix japonica*) within social groups^21,22^, we explore the effect of the sampling interval used and how behavior is defined based on topography and/or function^10^ on the distribution of events. We assessed not only Probability Distributions (PD), which provide information regarding how likely is an event of a given duration to occur, but also Relative Distribution (RD). These RDs provide information on how frequently an event would be recorded by the observer. In this analysis, longer events have more weight than shorter events, highlighting the representativeness of events within the observed animal’s time budget. We show not only that behavioral events last predominately less than 300 ms, but also, the impact of an insufficient sampling interval on distributions (PD and RD). In addition, we observe that power law distributions are widespread in the PD of event durations of behaviors that do not imply spatial displacement (e.g. vigilant, standing). We end by proposing a theoretical link between different behavioral distributions. In all, by combining the information on both PD and RD distributions and considering fine-grained time scales, we provide a framework to advance towards a statistical understanding of the diversity of types of behavioral events and the impact of the duration of the sampling interval in quantifying the events.

## Results

**Figure 1:**
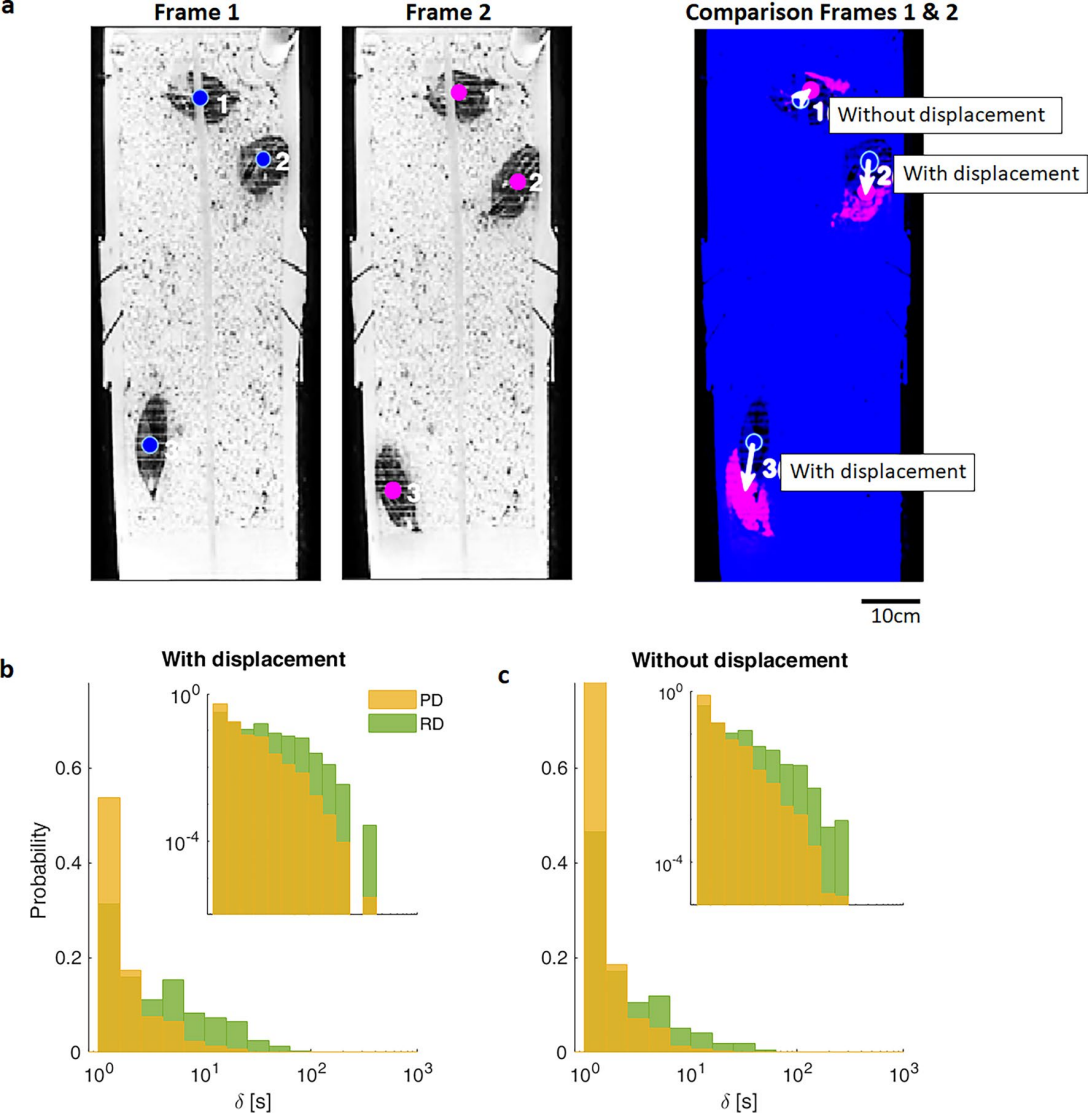
Statistical representation of events with and without spatial displacement in Japanese quail within their home-box social groups (Experiment 1). (**a**) Representation of the calculation associated with the definition of events with and without spatial displacement. Two consecutive frames are compared and the distance between the center of each animal (colored circles) is estimated (indicated by arrows in the third image). When the center of the animal moved more than 1 cm during the sampling interval, the animal was considered to be performing a spatial displacement. Oppositely, if the distance of movement was less than 1 cm, then it was considered without spatial displacement^22^. (**b**,**c**) Probability (PD) and relative distributions (RD) histograms of the duration (δ) of events with and without spatial displacement (**b**) and (**c**), respectively). Semilog axes are used and event durations (x-axis) are expressed in seconds, thus noteworthy histograms use logarithmic binning (x-axis), hence bin width increase logarithmically. Yellow bars belong to PD, estimated by counting the number of events that fall in the bin, divided by the bin width and the total number of observations. These PD show that there are many events of short duration and fewer long-termed ones. Relative distributions, depicted as green bars, are the total number of observations in each bin divided by the total number of observations. Note that the histograms start at δ =1s which also corresponds to the 1s sampling interval used herein. Insets show the same plot, but with a double logarithmic scaled axes (log-log).

In our first experiment, published^21,22^, and publicly available^23,24^ 1 h long, behavioral time series of Japanese quail within social groups (2 females and 1 male) were studied. Figure 1a illustrates the of two mutually exclusive behaviors with and without spatial displacement, based on an empirically established threshold value of 1 cm^22,25^. The Probability Distribution (PD) associated to the frequency of the occurrence of these events of duration δ for all animals is depicted in Fig. 1b,c. The semilog plot emphasizes the distribution of data over the broad range of temporal scales from 1 to 230 s, spanning more than two orders of magnitude. The insets show the same data in a double logarithmic plot. Note that, contrary to what would be expected for a normal distribution, the distributions present a slow monotonic decay with a predominance of short events. Hence, the distribution does not develop a middle peak associated with a mean duration value (δ) representative of the most frequent event duration.

Since PDs represent the frequency of occurrence of an event of a given duration on a logarithmic scale, the area of all bars must sum up one. Hence, the height (*h*_*i*_) of the *i*-th bin is computed as the number of events that fall in the bin (*c*_*i*_), divided by the bin width (*w*_*i*_) and the total number of observations (*N*). Mathematically it reads ${\text{h}}_{{\text{i}}} = \frac{{c_{i} }}{{w_{i} N}}$, which is consistent with $\frac{1}{N}\sum\nolimits_{i} {\frac{{c_{i} }}{{w_{i} N}}} = 1$, the normalization condition. But, for the logarithmic binning used herein (x-axis), bin sizes are not constant but rather increases logarithmically (see justification in “Methods” section “Logarithmic binning”). It is evident in the PDs show in Fig. 1 panels b and c that long events are less probable to occur than short events. However, since long events span over a longer period of time, they proportionally represent more of the actual time budget of the animal, than many frequent very short events. These long events may also be easier to be observed by the experimenter than short events. Thus, we propose complementing PD with Relative Distributions (RD) in order to describe the probability of observing events considering both, their frequency and their duration. Basically, in RD the height of a given bin becomes relative to the heights of its neighbors ${\text{h}}_{{\text{i}}} = \frac{{c_{i} }}{N}$, deprecating the bin width. Thus, in the RD histograms (green bars in Fig. 1b,c), the probability of long events is larger than in PD (yellow bars in Fig. 1b,c). In addition, consistent with the PD, these RDs also show a non-Gaussian type distribution.

**Figure 2:**
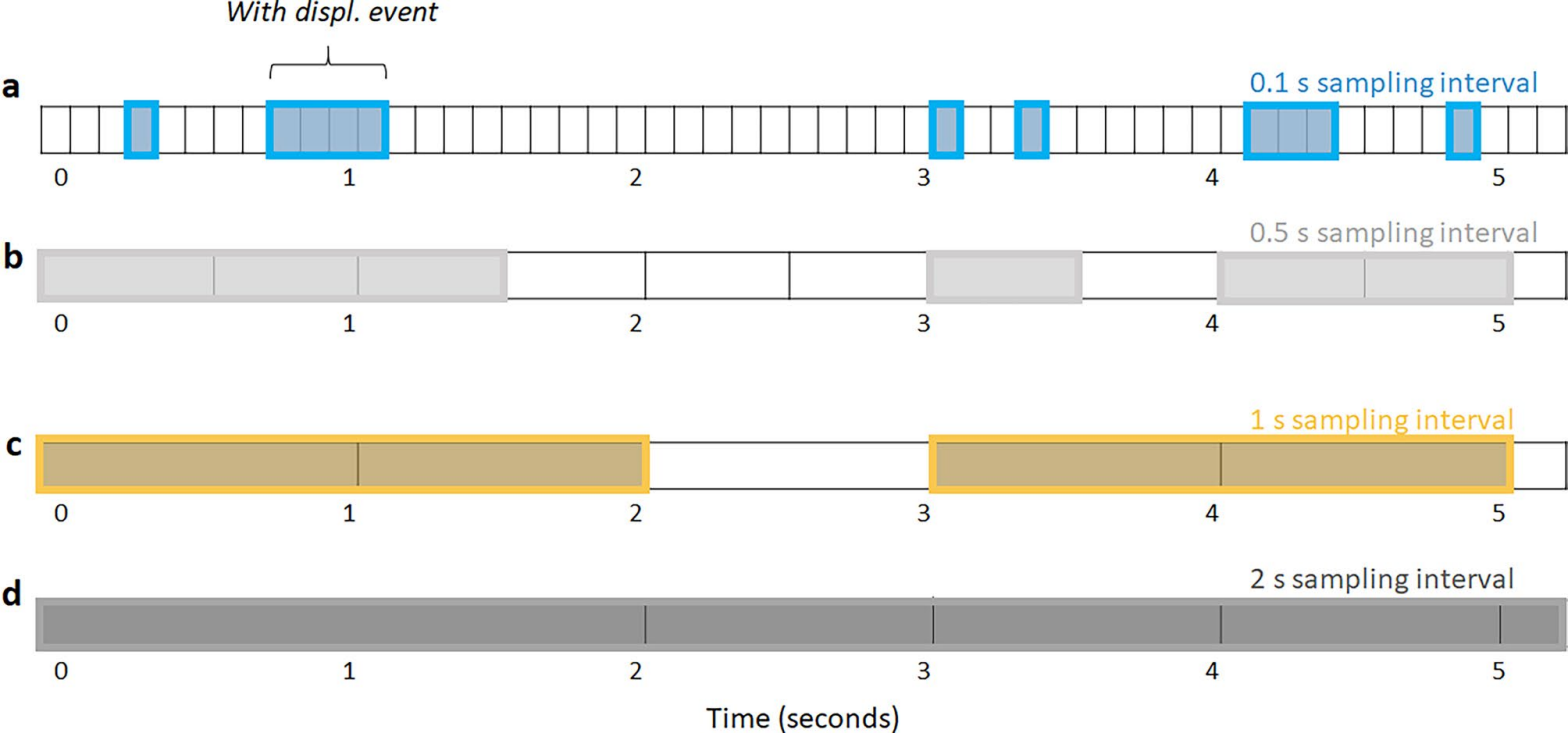
Schematic illustration of the impact of sampling interval on the duration of mutually exclusive behaviors. Each line represents progressively larger sampling intervals as follows: (**a**) 0.1 s, (**b**) 0.5 s, (**c**) 1s and (**d**) 2s sampling interval. Black horizontal lines mark sampling intervals, colored boxes represent intervals in which spatial displacement is performed within the sampling interval, while white boxes indicate periods without spatial displacement. Note that when the sampling interval increases (top-down), the events with spatial displacement appear to have longer durations, while the without spatial displacement (inter-) events go undetected. Upper brackets provide examples of the two types of mutually exclusive events.

It is important to remark that the histograms reported in Fig. 1b,c start at an event durations, δ, of 1 s which corresponds to the sampling interval used. Thus, any shorter event, if observed, would be recorded as part of the smallest bin potentially resulting in the blurring of the real shape of the underlying distribution for short durations (left side of plots). Since the 1 s bin is the highest in Fig. 1, it can be assumed that the actual temporal resolution of behavior could be below the 1 s sampling interval used. If so, an important question arises; How does the sampling interval length affect the distribution of behavioral events? To illustrate this, we show in Fig. 2 the event durations that would be obtained at four different sampling intervals. In this example, colored boxes represent intervals with spatial displacement, while white boxes represent time periods without displacement behavior (given the empirically established of 1 cm threshold value ^22,25^, as shown in Fig. 1a). Note how the same two mutually exclusive behaviors, when collected at the different sampling intervals, results in differences in the number and duration of registered events.

At a one-tenth of a second sampling interval (Fig. 2a), six events with spatial displacement are detected. Since with spatial displacement was defined as movement above the threshold during any point of the sampling interval, if sampling is increased five-folds (Fig. 2b) the first two events are merged into a longer one. Consecutively larger sampling intervals (Fig. 2c,d) lead to longer with spatial displacement events. Contrarily, given that events without spatial displacement can be considered as an inter-event (i.e. the period of time between with displacement events), when the sampling size is sufficiently large, short events seemingly go undetected as observed progressively from top to bottom in Fig. 2.

**Figure 3:**
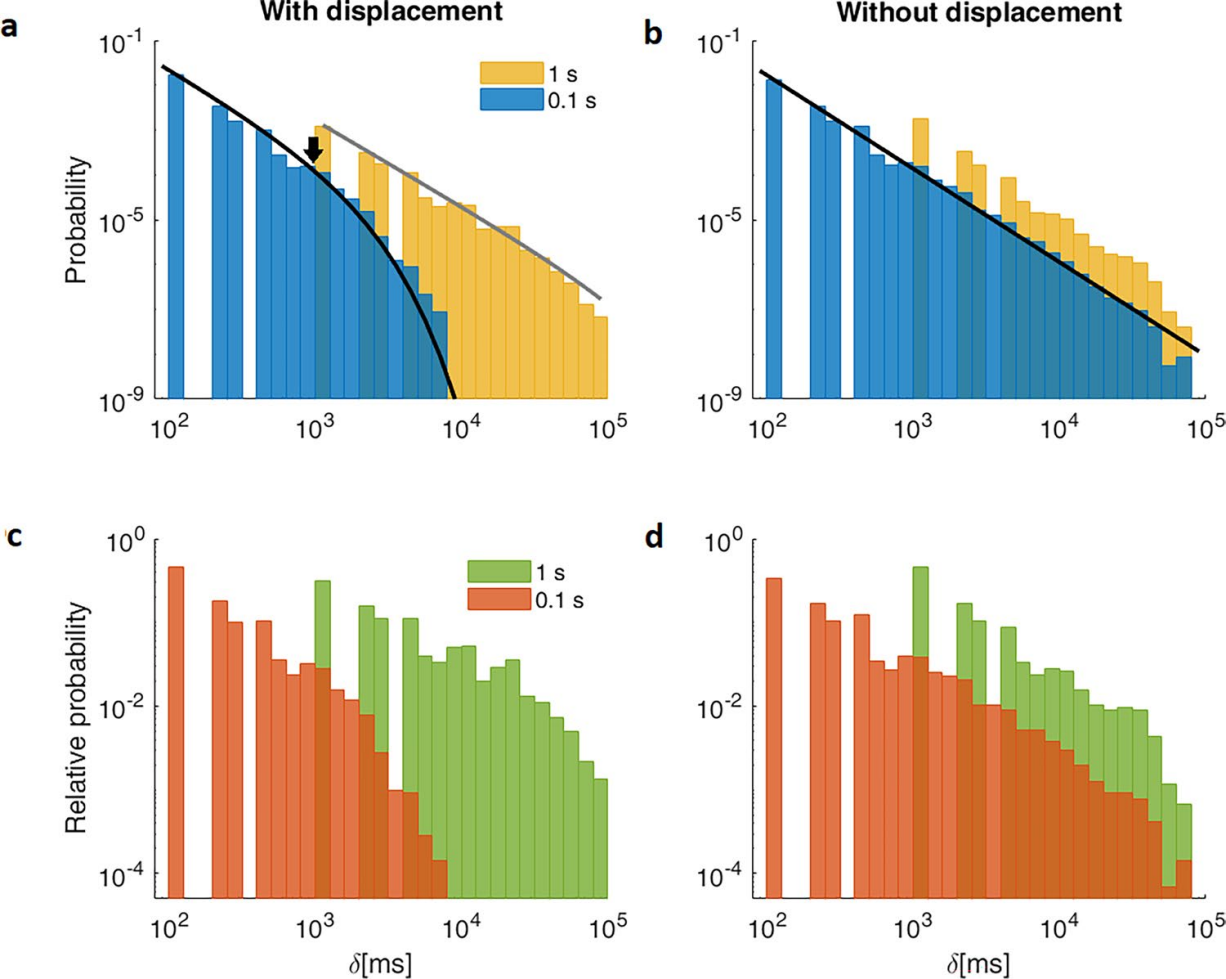
Differential effects of sampling interval on the distribution of events. (**a**,**b**) Probability (PD) and (**c**,**d**) relative distribution (RD) histograms for with displacement (left panels) and without displacement (right panels) events. The larger sampling intervals (1s) are depicted in yellow and green (as in Fig. 1), while the shorter sampling interval (0.1s) is shown in blue and red. In the case of with displacement events, the overestimation of the duration of the events becomes evident for the 1s sampling interval in comparison to the 0.1 s interval. Lines denote the fitting of the respective histograms (see Supplementary Note S1 for details), the black arrow marks the exponential cutoff observable for with displacement events at a 0.1 s, but not at the 1s sampling interval. Compare with a histogram from a single individual in Supplementary Figure S1. PDs are estimated by counting the number of events that fall in the bin, divided by the bin width and the total number of observations, while RDs represent the total number of observations in each bin divided by the total number of observations.

In order to visualize the effect of sampling rate on the PD of event duration on real data, we compare the same data time series shown in Fig. 1 sampled at both 0.1s and 1s intervals (Fig. 3). Note that the x-axis is now expressed in milliseconds to highlight the high-resolution time scales assessed.

In the PDs of duration of events with spatial displacement, this ten-fold decrease in sampling interval results in a dramatic shift to shorter event durations. When PDs were fitted (see details regarding fitting in Supplementary Note 1S), the resulting *probability density function*, *pdf*, shows a clear exponential cutoff (black line, Fig. 3a). According to fitting, this fast exponential decline starts at the characteristic time ~850 ms (black arrow, Fig. 3a). On the other hand, for 1s sampling rates, the estimated cutoff is not observable, presumably beginning beyond the range of the histogram. Hence, the PD appears almost linear on this double logarithmic plot (gray line, Fig. 3a), and just the power law-like behavior is observable. Similar effects of sampling intervals are appreciated in Fig. 3c where RDs are depicted. In other words, for with displacement events longer sampling intervals result not only in a gross overestimation of distributions but also an overall distortion of the shape of the PD and RD.

The contrasting effect of the resolution is observed for without displacement events, where shorter events are now evident, but the range and shape of the PD (Fig. 3b) and RD (Fig. 3d) are practically not affected by the ten-fold decrease in sampling interval used. Note that for both resolutions a linear behavior is observed on the double logarithmic scale (Fig. 3b) which is typical for power law at least up to the 100.000 ms assessed herein. Cumulative probability distributions are shown in Supplementary Fig. S2.

**Figure 4:**
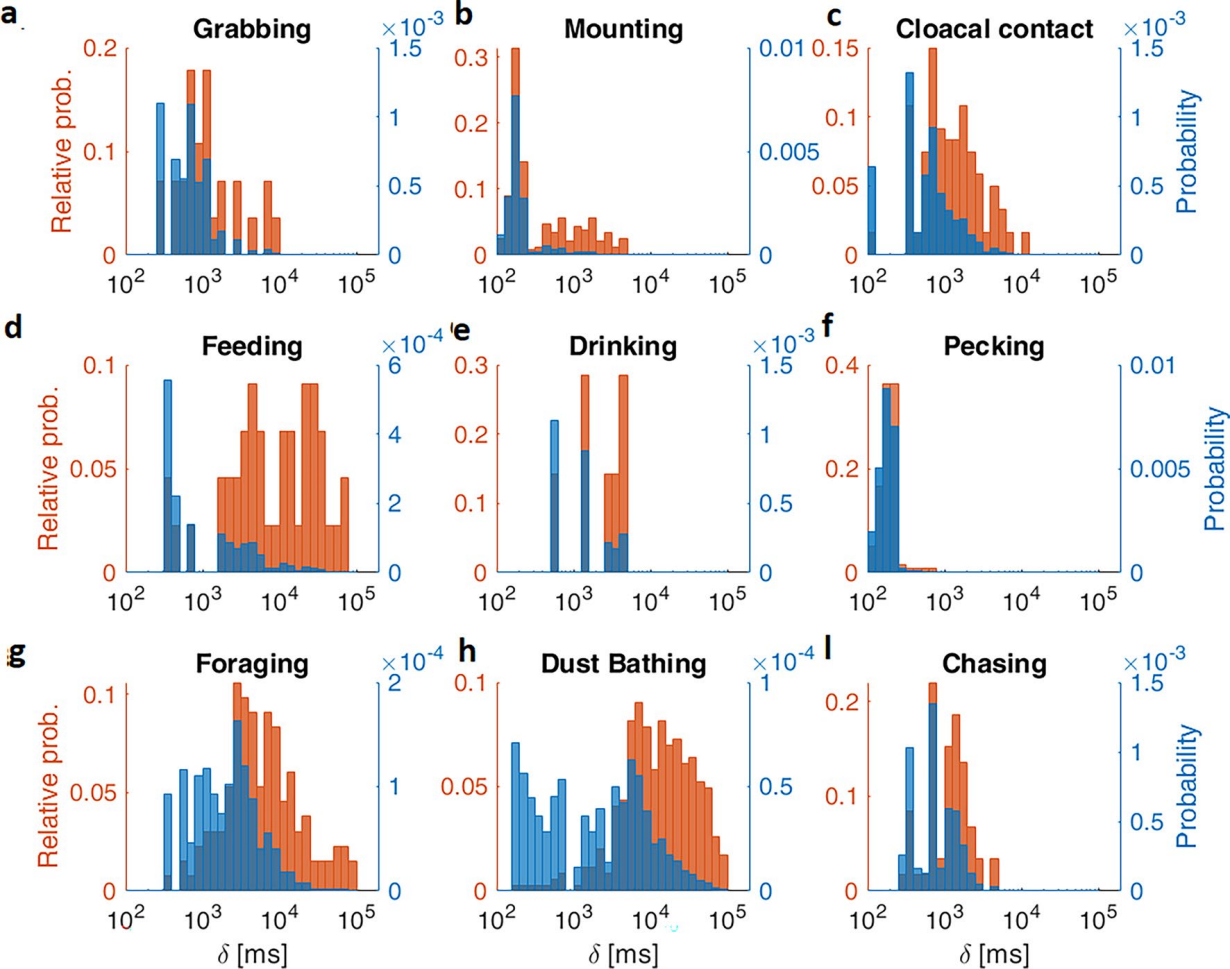
The shape of probability and relative distributions (PD and RD) of event durations (δ) depend on the behavior analyzed. Logarithmic histograms of the event duration of: (**a**) grabs, (**b**) mounts, (**c**) cloacal contacts, (**d**) feeding, (**e**) drinking, (**f**) pecking, (**g**) foraging, (**h**) dust bathing and (**i**) chasing. behaviors of male Japanese quail PDs are depicted as blue histograms whose scales are on the right-blue axes. Orange RD histograms represent the probability of finding an event that lasts the represented time-interval δ, and the corresponding scales are on the left, orange axes. Note, as in Figs. 1 and 3, that long termed events have a larger probability to be observed than the shorter ones (orange maxima) but most of the behaviors are short-termed (blue maxima).

How the dynamics of different types of behaviors are reflected in the probability and relative distribution of events is explored in Fig. 4. Here visual analysis of video recordings in real time was used to collect feeding, drinking, foraging, dust bathing, pecking, mounting, cloacal contact, chasing, and grabbing event durations by pressing keys in ANY-maze (see “Methods”, and definitions in Supplementary Table S1^22^). The resulting histograms are depicted using semi-log scales, where PD and RD are shown in blue and red, respectively. The characterization of these distributions is summarized in Supplementary Table S2. PD and RD were similar independent of sex and social group (Supplementary Figs. S3 and S4, respectively).

Figure 4 shows behaviors defined not only on topography but also including functional aspects^10^. Here, the shape of the PDs and RDs has dramatically changed compared to the definitions based on a threshold for movement as previously shown in Figs. 1 and 3. Most behaviors show distributions with a well-defined maximum value, indicative of a representative, behavior-specific, event duration (Fig. 4). Characterization of these maximum values (Supplementary Table S2), shows that for 4 out of the 9 behaviors analyzed, event durations below 300s are the most frequent (i.e. grabs, mounts, feeding and pecking). Differences between behaviors in regard to the range of possible values in event durations are also observed. In Fig. 4 only feeding, foraging and dust bathing (Fig. 4d,g,h respectively), and to a lesser extent cloacal contacts (Fig. 4c) show event durations spanning for more than one order of magnitude with appreciable probability (see also Supplementary Table S2). As a consequence, their PDs are shifted with respect to their RDs, indicating that although short events are more probable, longer events contribute proportionally more to the time budget (i.e. total time performing the behavior). This shift is most noticeable in dust bathing, given that it has the broadest range of durations, covering three orders of magnitude from 100 to 100,000 ms (equivalent to 0.1–100s; Supplementary Table S2). Moreover, their histograms appear to be bimodal (Fig. 4h, Supplementary Table S2), which could be associated with the fact that dust bathing involves a sequence of actions^26^.

The rest of the behaviors span for less than one order of magnitude (Supplementary Table S2) with appreciable probability (Fig. 4a,b,e,f,i). For these behaviors, the probability of relatively short or large events is low in comparison with that of the representative peak duration. Hence, bins of both PD and RD distributions have widths of the same order of magnitude (x-axes) and almost the same number of events will fall on each one (y-axis), thus the shape of both distributions almost match.

**Figure 5:**
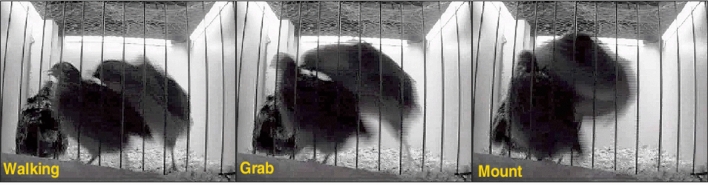
Representative example of high-speed social interactions between Japanese quail within social groups. Three consecutive frames are shown in which different behaviors are observed. In this example, the grab event only lasts 1 frame, hence presents a duration of 66ms.

**Figure 6:**
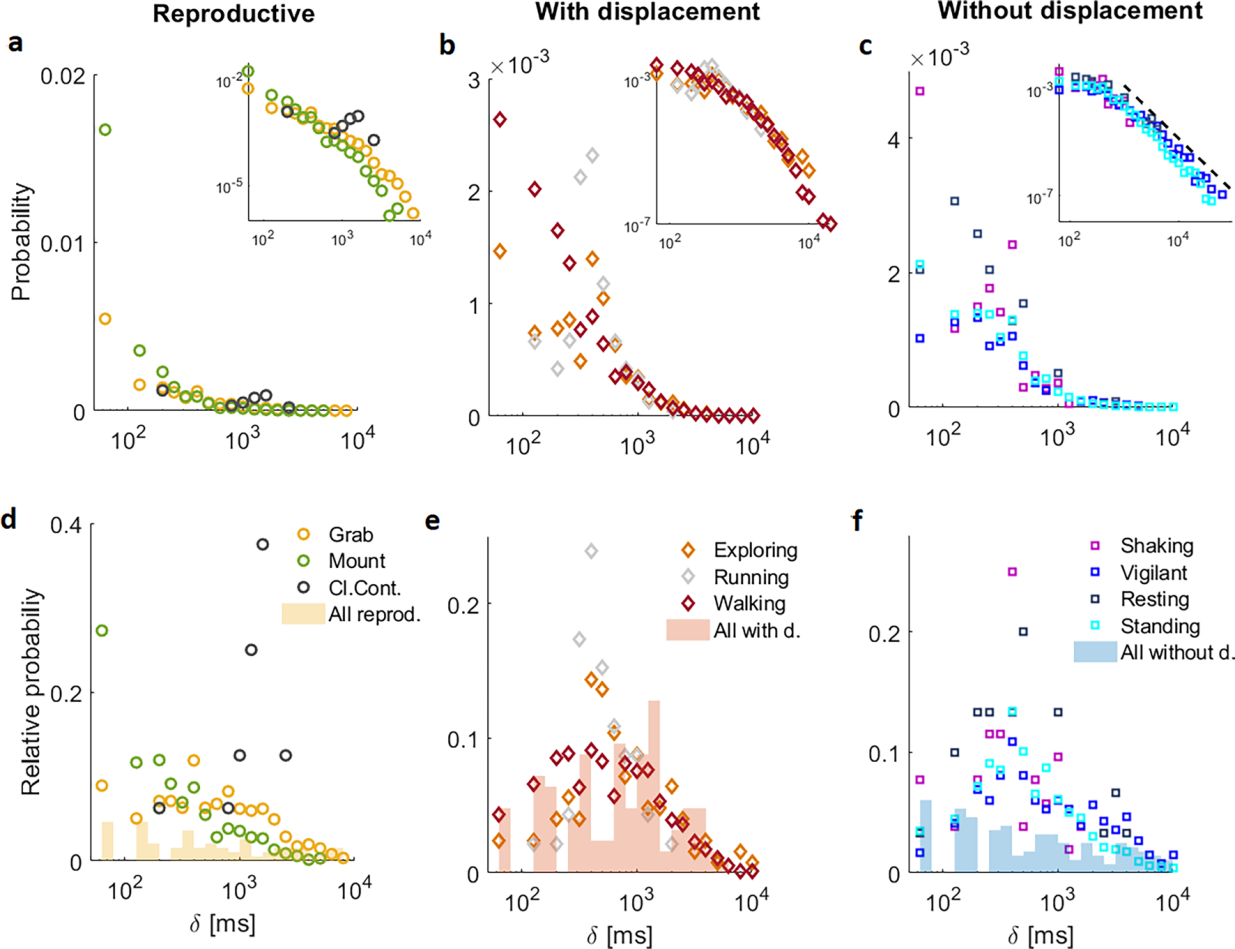
High-resolution sampling and fine-grain behavioral definitions highlights behavior-specific temporal organization as shown by the analysis of the corresponding probability and relative distributions (PD and RD) of event durations. (**a**–**c**) PD semi-log plots and (**d**-**f**) RD semi-log plots of behaviors grouped into three general categories: (**a**,**d**) reproductive, (**b**,**e**) behaviors with spatial displacement and (**c**,**f**) without spatial displacement. Insets in panels (**a**-**c**) show PD using a double logarithmic scale, where the drawn dashed line in the panel c inset has a slope of -2, and has been provided as a visual reference. See numerical characterization in Supplementary Table S3. In panels d-f solid bars represent the overall histogram if all the events from each behavior depicted in the panel were combined.

To further characterize the time interval in which different behavioral events are performed, a 10 min high-resolution data set was used (see Experiment 2 in “Methods”). Although this data set is of shorter duration than the previously shown, it has several advantages. First, in addition to the top camera, a side camera was included that allowed enhanced visualization of behavior. Second, a high-resolution ethogram was used to record the male´s behavior at a frame-by-frame resolution (sampling interval of 66ms), as exemplified in Fig. 5. Third, the experimental design favored the performance of high-speed social behaviors. Specifically, a socially isolated adult male Japanese quail was introduced into a novel group of females. This experimental design allowed exploration of the functional relationships between different behavioral definitions in regard to their probability distributions, as well as assessment of appropriate behavior-specific sampling intervals for the study of social interactions in Japanese quail. The number of events of each type of behavior is shown in Supplementary Table S3. The most frequent behaviors are shown in Fig. 6, with PD and RD represented on semi-log scales. As in the previous experiment (see Fig. 4), differences in distribution are observed between behaviors. Reproductive events, grab and mount, predominately last only 1 frame (i.e. 66 ms, Fig. 6a), thus, a characteristic duration could not be determined even for this high-resolution sampling interval, since it was insufficient to characterize the lower limit of the distribution. These fast changes between behaviors was most likely associated with the particular experimental design which involved an unfamiliar male. Contrarily, cloacal contacts did present a characteristic duration, as observed by the maximum probability value at ~200 ms (Supplementary Table S3) in both PD and RD (Fig. 6a,d, respectively). Moreover, log-log plots (Fig. 6a, inset) do not show linearly but rather an increasingly faster slope with increasing durations, representative of an exponential cutoff.

In regard to behaviors that involve spatial displacement, walking events predominately last only 66ms but spanned over a broad range up to 10^4^ ms (Fig. 6b, Supplementary Table S3). As with reproductive behavior, an exponential cutoff in the PD is observable in log-log plots (Fig. 6b, inset). The corresponding RD of walking is also broad, but a maximum value is observable at ~400 ms (Supplementary Table S3). The exploring behavior showed a similar distribution as walking behavior, while running presents a characteristic maximum duration value at ~400 ms and a more limited range of durations spanning only 1 order of magnitude, as observed in both PD and RD (Fig. 6b,d, respectively; Supplementary Table S3).

**Figure 7:**
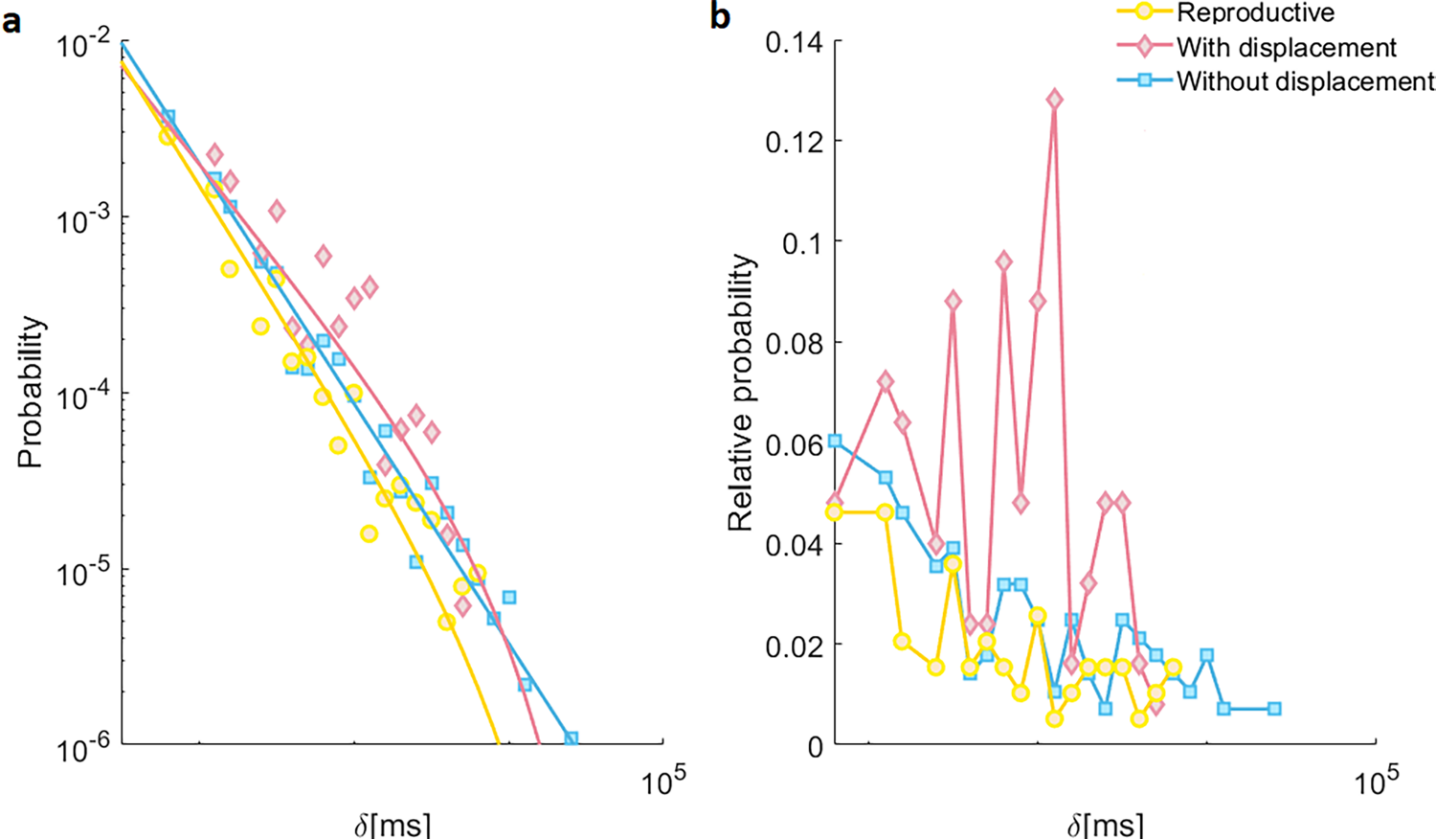
Probability and relative distribution of reproduction, with displacement, and without displacement events obtained by integrating associated behaviors. (**a**) PD on a double logarithmic scale shows a monotonic decreasing behavior. Since very short events are the most probable, they still range from 60ms. Note that the three distributions can be modeled by the PL+Exp function. However, reproduction and with displacement present clear cutoffs, while immobility does not. The continuous lines are fittings, note that reproduction and with displacement have a cutoff missing in without displacement behavior. (**b**) Corresponding RDs. Without displacement behavior has lost the mean value (peak) and is monotonic decreasing. Time series were approximated using the same data time series analyzed in Fig. 6.

**Figure 8:**
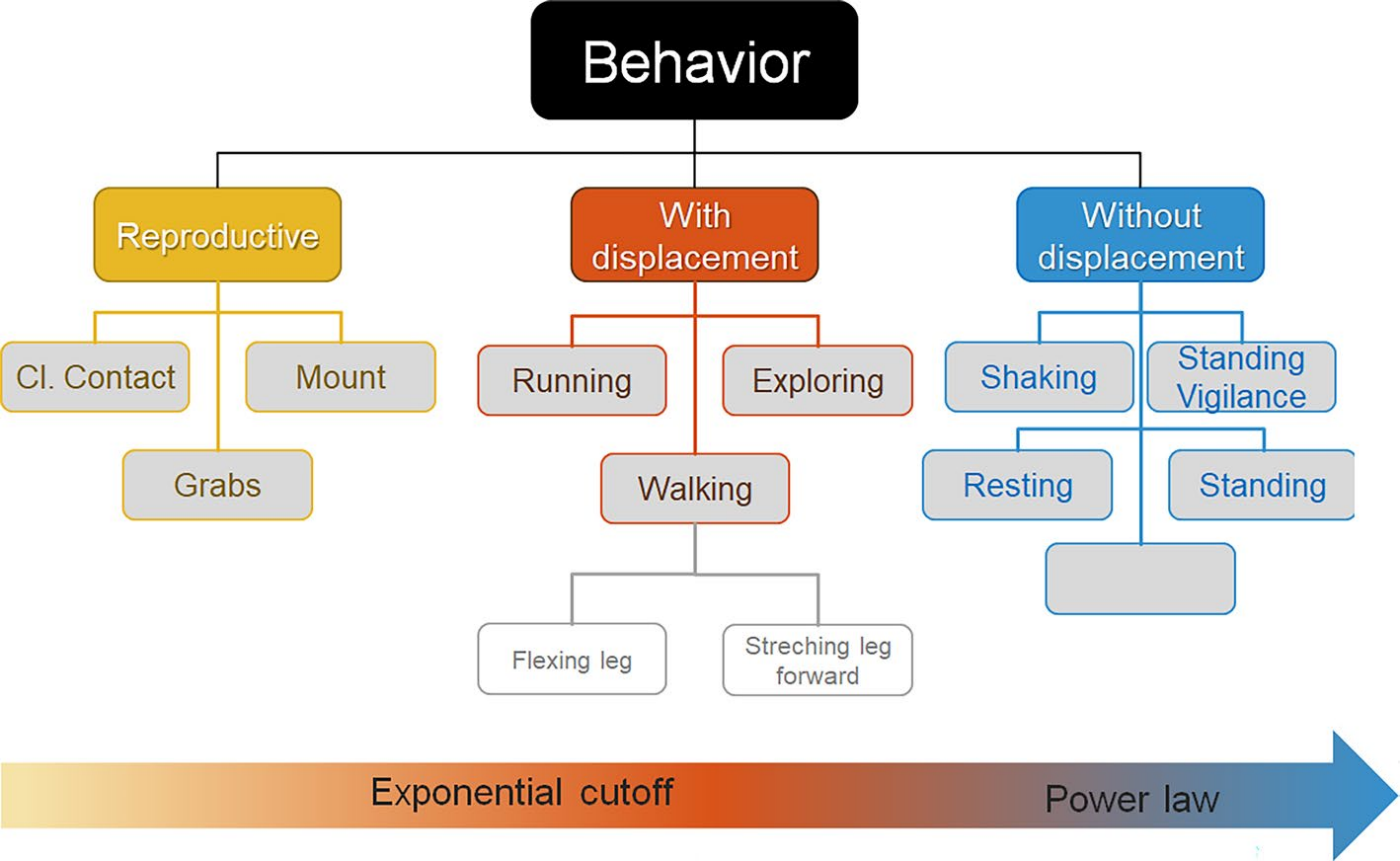
Hierarchical model of association between behavioral definitions. Lower levels fine-grain definitions can be considered as finer details, and thus can be integrated into coarser-grained definitions conserving characteristics associated with their distributions. Categorical representation of the behavior definitions.

In regard to behaviors without spatial displacement, the behavior with the broadest range of scales is standing vigilant (three orders of magnitude; Supplementary Table S3). Moreover, the corresponding log-log plot (Fig. 6c, inset) shows that the tail of its distribution reflects the mentioned power law distribution observed in the more general definition of events without spatial displacement previously shown (Fig. 1b, inset). Shakes are an interesting exception, while the behavior does not imply spatial displacement, they occur on a very fast time scale (Fig. 6c,f, Supplementary Table S3). From Fig. 6 it is evident the way behaviors are defined, as well as the sampling interval, significantly impact the statistical properties of the resulting temporal distributions. Hence, we continue by exploring how each of the fine-grained behavioral distributions contribute to the distributions of general coarse-grain behavioral definitions. We recreated the more general reproductive, with and without spatial displacement time series by integrating associated high-resolution behavioral time series based on whether they are associated with reproduction, involve spatial displacement or do not involve spatial displacement, respectively. In these recreated time series, event durations reflect a consecutive period of time while the Japanese quail is performing any of the encompassed behaviors (Fig. 7, Supplementary Table S3). The probability distributions of the recreated reproductive and with displacement events show exponential cutoffs (yellow and red, Fig. 7a), with the most frequent event being ~60 ms. However, RD shows a predominant maximum value at 1200 ms for with displacement events (Supplementary Table S3, Fig. 7b), indicating that longer events in the order of seconds are important contributors to the time budget of the animal with higher probabilities to be observed by the experimenter. Noteworthy, in the recreated without spatial displacement events, the expected power-law distribution (blue in Fig. 7a) on the double logarithmic scale is observed. This capability of recovering behavioral distributions from by grouping specific behaviors, can be understood through hierarchical links between the different types of definition of behavior. Such hierarchy is schematically represented in Fig. 8. At the top is the complete behavior set, which is trivially described by a single dot in the distributions. A step down our first classification, definitions such as reproduction, with and without displacement are presented. When a sufficient high-resolution sampling interval is used, they are shown to present exponential cutoff or power law PD as indicated by the bottom arrow. Stepping downwards, closer to the bottom, we have a more refined behavior description, representing more specific actions. Interestingly, most of the PD of these behaviors shown in the lower levels have the same type of distribution as the corresponding parent behaviors (compare Figs 6 and 7).

## Discussion

Herein we show that the 0.5s – 6 min sampling interval used previously to assess the temporal dynamics of behavior in small animals^18,27–31^ including Japanese quail^11–13,31,32^ is insufficient to capture the most frequent event durations. Many of the behaviors studied showed that the most probable event durations lied between 60 and 300 ms (Supplementary Tables S2 and S3). Moreover, maximum probability values in PD of event durations, for some behaviors, coincides with the sampling interval. Hence, it is logical to assume that the actual temporal resolution in which the animal is behaving and interacting with conspecifics could be faster than our 60 ms lower limit. This result is highly relevant since it reflects the Umwelt (self-centered world) of the Japanese quail, and its potential for perceiving and sensing its environment at these high resolutions. Although the concept of Umwelt is around 100 years old^7,33,34^, only more recently do researchers have readily available technology to study animal biology with an unprecedented level of temporal detail and across the full range of ecological scales^35,36^. In this modern technological landscape, the importance of the appropriate grain for characterizing behavioral dynamics and species-environment relationships is two-sided. On one side of the spectrum, if we continue making studies from an anthropocentric perspective^6^, which could lack sufficient sampling resolution, many behaviors and/or fundamental signals may go unrecognized. On the other side, when using arbitrarily high sampling resolutions above the appropriate grain, data may become noisy, thus difficult to interpret and lose relationship with its function^10^.

Traditionally, animal behavior is analyzed in real time. Thus not only is it recorded on a human perception temporal scale, but also, may be biased by how different types of behaviors are perceived by the human brain. The human perceptual system is strikingly able to detect and recognize the motion of dynamic stimuli^37,38^. Thus, in most studies, with obvious exceptions such as sleep studies, if during a sampling interval an active behavior with spatial displacement is performed, it would be recorded instead of the less notorious inactive pauses. As shown in Fig. 2, a biased focus on activity has enormous implications. It leads to a gross overestimation of the duration of active events, a loss of information regarding short inter-events, as well as distortions in the shape of the probability distributions. This last point could lead to misclassification of a distribution as a power law solely due to an insufficient sampling interval. Different approaches to coarse-graining also could impact differentially the resulting shape of the PD and RD, Thus, in future studies would be necessary to elucidate the effects on PD and RD of different coarse-graining methods, for example recording the behavior performed for the most time during the sampling interval (i.e. the mode).

By combining the use of both PD and RD we were able to provide a novel perspective in regard to the statistical distribution of behavioral events. Moreover, our results are self-consistent, and extend upon the understanding of behavior as the output of a large nonlinear dynamical system (e.g. the brain-body-environment ensemble) whose repertoire includes the diversity of dynamics observed in our experiments^18^. Using high-resolution data (Figs. 4 and 6) we show that, in general, behaviors can be qualitatively grouped into 3 conspicuous types. The first type corresponds to behaviors with evident peaks in both PD and RD distributions (although not always observable at the resolution evaluated herein), and that span over less than an order of magnitude. Hence, PD and RD overlap, e.g. pecks or shakes. The second type of behaviors have characteristic durations, but span over several orders of magnitude, e.g. walking, and exploration. Exponential cutoffs are observable at time scales in the order of seconds-minutes. Their respective PDs and RDs do not overlap, but rather show complementary information. The third type of behaviors show a broad range of possible durations spanning over three or more orders of magnitude, e.g. standing. RD shows higher probabilities of larger event durations and wider distribution in comparison to PD. For these last behaviors, linearity in log-log plots PD is observable, indicative of a power law type distribution. This diversity in types of observed behaviors highlights the importance of not assuming a typical mean value, i.e. the probability of a given behavior as normally distributed, as well as the need to consider the actual distribution in the behavioral events under analysis.

Our in-depth look at the high-resolution data, also revealed differences in regard to the two datasets evaluated revealing insight on the impact of experimental design on PDs. Specifically, in regard to social behaviors, while the shape of the PD differ between experiments, in both the most frequent duration of grabs, mounts, and pecks observed are very short, being as low as 66ms (Figs. 4 and 6). Hence, the fast speed of social interactions does not hinder the detectability of the event in real-time^39^. Although, social cues included in short pauses or fast transitions between different types of social behavior may go unrecognized when videos are analyzed in real time in comparison to high-resolution frame-by-frame analysis. Contrary to the short durations of social interactions, dust baths, when present, show a very wide distribution with at least two peaks around 200ms and 7000m. (Fig. 4, Supplementary Table S2), which could reflect the fact that it involves performing a specific sequence of components^26^. Hence, the first maximum value observed in the distribution may reflect the characteristic duration of the sequence. The following maximum values in the distribution may reflect the number of repetitions of the sequence or different levels of completeness. Dust bathing in this sense is not unique, even behavior definitions such as walking could also be further reduced to a repetitive sequence of individual components associated with leg movements. Hence, even higher resolution datasets would be useful to further characterizer the association between the sequence of movements that are associated with a given definition of behavioral event (see also model Fig. 8).

In both datasets, events without spatial displacement show power law type distributions. Different observations of such systems often follow a similar profile across multiple measurement scales (‘‘scale-free’’ patterns). As a consequence of their non-Gaussian distributions, event durations are not well characterized by a typical or average value^40^. Naturally, there are biological limitations regarding the maximum possible event durations, which is usually mathematically modeled as an exponential cutoff of the power law distribution^41,42^. Guzman et al.^11^ from a week-long study with a 0.5 s sampling interval, showed that these inter-events can be as long as 1h and present a power law like distribution at least up to 250 s. Contrarily, these authors showed that with displacement events always lasted less than 40s and presented a sharp exponential cutoff (see Supplementary Material in Guzman et al.^11^). This difference in the distribution, between with and without displacement events could be attributed to the energetic cost of movement^43,44^. Japanese quail are considered a polyphasic animal, with periods of sleep present throughout the daily 24 h period^45^. Hence, energy conservation and sleep, could both help to explain the long periods without displacement represented by the tail of the power law distribution.

Knowing when a variable does or does not follow a power law provides important theoretical clues about their underlying generative mechanisms^40^. It can also facilitate statistical extrapolations about the likelihood of very large events^46^. Chu-Shore et al.^47^ argue that, in humans, sleep-wake stage distributions are often complex, thus distinguishing between exponential and power law processes may not be straightforward. For example, multi-exponential distributions can also appear linear on a log-log plot^47,48^. Specifically, if the part of the distribution corresponding to short time scales is obscured it is particularly difficult to distinguish a power law pattern, from alternative heavy-tailed distributions like stretched exponential or log-normal^40^. The advantage of our current study, in comparison to previous work in the field, is the high-temporal resolution of data. Although the two experiments analyzed were too short (≤ 1h) to observe long-durations of events that are needed to adequately approximate the tail (right-side of plots) of power law distribution, the upper part of the distribution (left side of plot) were observable for the first time. After collecting and analyzing over 6000 behavioral events we show that linearity in the log-log plot of the duration of without spatial displacement events holds up to 66 ms (Fig. 3b). Consistently, other analysis methods, such as Detrended Fluctuation Analysis, have shown long-range auto-correlations in a variety of behaviors, associated with long-term memory^11–13,18,30^^,^^31^. Interestingly, herein not only without spatial displacement showed a power law distribution, but also the behaviors included within it (Figs. 6c and 7). It is unlikely that these distributions can merely stem from a sum of exponentials^47^, associated with superimposed behaviors. It is more likely that without spatial displacement captures the emergent complexity and correlation properties of the system. Hence, a hierarchical organization of the behavior can be established according to the behavior definition (Fig. 8).

How to define behavior and how to determine its duration is commonly associated not only with the objectives of the study but also with practical limitations such as how to conveniently position the camera, the sampling effort, etc. From our in-depth analysis of high-resolution behavioral time series, we can conclude that using a sampling interval shorter than the speed in which animals transition into and out of the behavior has important consequences. Not only because the duration of the activity events will be severely overestimated and the shape of the distribution could be distorted, but more importantly, because there are social interactions that may go unrecognized. The observation of the fast speed at which Japanese quail behave highlights the importance of moving away from anthropocentric ways of measuring behavior and redefining events based on the temporal scale pertinent to the species under study using an organism-centered perspective. Moreover, probability distributions of event durations vary between behaviors, thus we propose a hierarchical model that links diverse types of behavioral definitions and distributions. From this model it is evident that power laws do not emerge from a sum of exponentials but rather as an emergent property of the system, at least up to the time scales studied herein. These findings not only pave the way towards a statistical framework for defining behaviors but also have applied relevance, for instance, in the study of behavioral dynamics in diverse fields such as animal welfare, social dynamics, habit selection, and behavioral ecology. By ensuring the right species- and behavior- specific time scale we will advance towards a better learning and hopefully a better interpretation of how animals interact with conspecifics and their environment.

## Methods

### Animals and husbandry

The study was performed with the Japanese quail (*Coturnix japonica*), a species widely used for covering neuroendocrine and social behavior studies^49,50^. The animals were bred according to standard laboratory protocols^22,25^. The experimental protocol was approved by the Institutional Council for the Care of Laboratory Animals (CICUAL, Comité Institucional de Cuidado de Animales de Laboratorio) of the Instituto de Investigaciones Biológicas y Tecnológicas (IIByT, UNC-CONICET). Animal care and experimental treatments followed the Guide for the Care and Use of Laboratory Animals issued by the National Institute of Health (NIH Publications, Eighth Edition)^51^. They also followed local animal regulations including the Animal Protection law number 14346, National Administration of Drugs, Foods and Medical Devices (ANMAT) decree 6344/96, and the National Scientific and Technical Research Council (CONICET) resolution number 1047/2005. This study was carried out in compliance with the ARRIVE guidelines.

### Experiment 1: behavioral dynamics of Japanese quail within their social environment

This experiment as well as the data time series obtained have been thoroughly described previously^21,22^. The effect of social environments and the consequence of dominance at an individual level on the temporal dynamics of behavior has been assessed in Alcalá et al.^21^. Briefly, a total of 106 Japanese quail (53 females and 53 males) were subjected to a combination of 4 preselection tests to favor diversity in the type of social groups. This was based on taking into consideration that Japanese quail that are more fearful also tend to be more aggressive^52^. Then, social groups (2 females: 1 male) of animals (156-171 days old) were housed in a white wooden apparatus measuring 80 × 40 × 40 cm (width × length × height, respectively) with wood shavings on the floor. A feeder and an automatic nipple drinker were positioned in opposite corners of the apparatus. Monofilament nylon lines were extended over the top of the boxes with a 1 cm separation to prevent the birds from escaping but without interfering with their visualization. A closed-circuit television (CCTV) video camera (Pronext, EX20 4N1 1.0 HD) was suspended 1.5m above the box. After a 2-day habituation period, idTracker^53^ in MATLAB R2017a was used to register the position of each animal at each frame (15 frames per second) during a 1 h period. Behavioral data were recorded in the form of a time series of mutually exclusive states. In the case of the with spatial displacement (locomotor) time series, at any given time, if the bird moved over the 1 cm threshold during the sampling interval (i.e. 0.1 or 1s) a number one was recorded, or zero if immobile. Datasets are publicly available at figshare^24^.

Time series of non-ambulation behaviors were obtained through visual observation of video recordings using an interface ANY-maze to register behavior. Definitions have been provided in Supplementary Table S1. For each bird, when the specific behavior was performed the corresponding key was pressed until the bird finished performing the behavior, thus a binary time series, x_i,_ sampled at up to 2 data points per second was constructed for each behavior. Where, x_i_ = 0 indicates that the animal is not performing the behavior, while x_i_ = 1 indicates that the animal is performing the behavior, time series are publicly available at figshare^23^.

### Experiment 2: high-resolution dynamics of male Japanese quail associated with the display of reproductive behavior

This study is part of a larger experiment designed to assess the capability of accelerometers to detect reproductive behavior in male Japanese quail^54^. Raw data is publically available at figshare^55^. Thus, 2/3 of the birds (male and females) had accelerometers attached as a dorsal patch or a backpack which, in general, did not interfere with social or reproductive behaviors^56,57^ All birds were subjected to the same manual manipulation in order to reduce variability between individuals. At the beginning of the experiment, 2 adult females (156-171 days old) were housed together in a white wooden apparatus measuring 40 × 40 × 40 cm (width x length x height, respectively), with 3 wooden walls and one wire mesh wall. After 9 days of habituation to the novel apparatus and social environment, an adult unfamiliar male was introduced into the box, and the behavior was recorded during a 10 min period.

Two closed-circuit television (CCTV) video cameras (Pronext, EX20 4N1 1.0 HD) were used to record behavior, one camera was suspended 1.5m above the box, and the second camera was positioned on the other side of the wire mesh wall. This second side camera allowed visual observation of the whole animal, and thus more specific behaviors were able to be recorded at the temporal resolution of the camera (15 frames per second, equivalent to 67 ms) in comparison to Experiment 1. Male behavior was recorded using a customized app in MATLAB R2017a which is available to download from figshare^58^, where each frame was observed and the behavior being performed was recorded. For each behavior a binary time series, x_i,_ sampled at a 67ms, the interval was constructed (9000 data time points).

### Data analysis

For each bird, behavioral events defined as the interval of time that an animal continuously performs a given behavior. In practice this consists in counting the number of continuous sampling intervals in the time series in which the behavior was recorded^11,12^. All events were organized as values of a vector, H, in order to perform the corresponding histograms.

#### Logarithmic binning

This is a simple way of smoothing sample data which is distributed on a logarithmic scale and allows the graph to convey information about how many events are in a given range. Logarithmic binning is specifically important for understanding the distribution of events with relatively long durations which can be so infrequent that traditional linear histograms plots become uninformative (for further discussion see^40,59^). Herein, a logarithmic scale was used for binning data to capture time scales ranging from the lower limit (tmin) of possible durations of the behavioral event associated with sampling frequency. The upper limit (tmax) is defined by the longest observed event of a given behavior. The distribution of the bins’ edges is defined through the vector B=10^[emin: 0.1: emax]^ in such a way that tmin=10^emin^ and tmax=10^emax^, and a step of 0.1. Note that the first bin will include all the events with durations between tmin and 10^(emin+0.1),^ such a feature is better illustrated in Supplementary Table 4 where, for each experiment, we present the values of emin and emax and the first and the last bin borders. Thus the first element of B coincides with tmin and the latest with tmax. With these logaritmic definitions of beginning, the distributions are straightforward obtained by the MATLAB function.

1 $$\left[ {{\text{Y}},{\text{X}}} \right] = {\text{histogram }}\left( {{\text{H}},{\text{B}},\text`{\text{normalization}}\text',{\text{norm}}} \right),$$

Were H is the vector containing all the recorded durations for a given experiment and behavior definition and B defines the binding. The parameter *norm* allows to specify the desired type of distribution. Its specification is built-in and agrees with the definitions presented in Supplementary Note S1. This parameter will take the value ‘density’ for PD, ‘probability’ for RD and ‘cdf’ for CD. This MATLAB function plots the corresponding histogram and saves the result in vectors X for the abscissa and Y for the height of the bars.

#### Fitting algorithms

In order to characterize probability distributions with a representative function, data was fitted with a power law function with an exponential cutoff in MATLAB, using the following MATLAB code.

$${\text{f}} = {\text{fit}}({\text{X}},{\text{Y}}, \text`{\text{a*}}\exp ({\text{ - x/b}}){\text{*x}}^{{\text{( - c)}}}\text' ),$$

The vector X contains the duration of the event and the vector Y the probability of observing an event of a given duration. These vectors are used as imput data to fit a continuous function that describe the distribution in a compact way. The third parameter corresponds to the definition of the objective function used to fit.

From such a definition, we identify the characteristic time for the cutoff as with the parameter *b* and the exponent of the power law *c*.

### Supplementary Information

Supplementary Information.

## Data Availability

The data from experiments 1 and 2 that support the findings of this study are publically available on figshare^24,55,58^ at the following links: https://doi.org/10.6084/m9.figshare.7117679.v1, https://doi.org/10.6084/m9.figshare.7117631.v1, https://doi.org/10.6084/m9.figshare.21792887.v1.

## Code availability

The study was performed using publically or comercially available code. The idTracker has been previously published^53^. Our customized MATLAB app for analyzing simultaneously side and top cameras is published on figshare^60^, at available at 10.6084/m9.figshare.21900423.v1. MATLAB code used for performing histograms and fitting is provided in the Methods section. The commercially available ANY-maze Video Tracking System software can be downloaded at www.anymaze.com.
