# Supplementary material for: The relevance of a right scale for sampling when studying high-resolution behavioral dynamics
Source: Sci Rep. 2023 Aug 16;13:13291. doi: 10.1038/s41598-023-39295-z (PMC10432462; doi:10.1038/s41598-023-39295-z)
Supplement: Supplementary file 1 — Supplementary Information. [file 41598_2023_39295_MOESM1_ESM.pdf]

Supplementary Information

The relevance of a right scale for sampling when studying high-resolution behavioral dynamics

Barberis, L.[1,2], Simien, C.[4], Marin, R.H.[3-4], Kembro, J.M.[3-4*]

[1] Universidad Nacional de Córdoba, Facultad de Matemática, Astronomía Física y Computación, Córdoba, Argentina.

[2] Consejo Nacional de Investigaciones Científicas y Técnicas (CONICET), Instituto de Física Enrique Gaviola (IFEG), Córdoba, Córdoba, Argentina.

[3] Consejo Nacional de Investigaciones Científicas y Técnicas (CONICET), Instituto de Investigaciones Biológicas y Tecnológicas (IIByT), Córdoba, Córdoba, Argentina.

[4] Universidad Nacional de Córdoba, Facultad de Ciencias Exactas, Físicas y Naturales, Instituto de Ciencia y Tecnología de los Alimentos (ICTA), Córdoba, Córdoba, Argentina.

* Corresponding author: Jackelyn M. Kembro (jkembro@efn.uncor.edu)

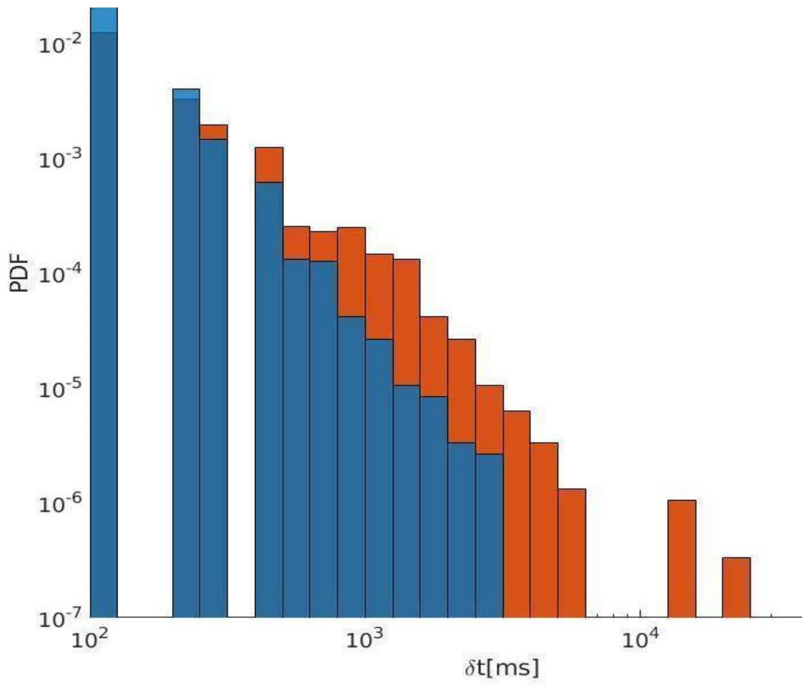

Supplementary Figure S1. Probability distribution of the duration (δ) of with (red) and without (blue) spatial displacement of a single individual sampled at 0.1 s. Note the overall similar shape as shown in Figure 3a and b for all birds combined. PDs are estimated as by counting the number of events that fall in the bin, divided by the bin width and the total number of observations.

Supplementary Note S1. Power law distributions and exponential cut offs

Power law distributions are mathematically described by the function $P(\delta) = a\delta^{-b}$ $P(\delta) = a\delta^{-b}$, and predict that it is possible to observe events of any duration, being the larger ones the less probable. Since real systems are not infinite in size and limited by both sampling resolution and the duration of the experiment; extremely large or extremely short spanned events are not possible. This is the case of distributions in Figure 2 (main text). Such distribution is expected to fall faster after some value. In practice, this is achieved by multiplying the Power Law by an exponential function. i.e. $P(\delta) = a.\delta^{-b}exp\left(-\frac{\delta}{\delta_c}\right)$. The value δ_c is a “characteristic value” that defines a “cut-off” that represents the largest event duration before the power law type distribution is abandoned. Interestingly, in the distribution for immobility events in Figure 2c, the exponential cutoff is not observed for the range of time scales evaluated. Hence, the cutoff for the power law most likely occurs at temporal scales larger than those evaluated herein, as has been observed previously in Guzman et al (2017)[1]. A complementary commonly used method for visualizing PL is the Cumulative Distribution (CD), which is provided for comparison in Figure S2. Just, note that linearity is also observed in the log-log plot of the CDF of durations of immobility events, providing further evidence of PL-type distributions.

To summarize, the power law behavior of the duration of events without spatial displacement is consistent with the previously described properties of long-range correlations and scale invariance (see main text for details). Specifically, scale invariance implies that the same pattern is observed independently of the temporal scale of observation. Hence, the sampling interval used (within reasonable limits) does not affect the scaling properties (i.e. linearity in the double logarithmic plot or slope) as observed in Figure 2C. Conversely, since the probability distribution of the duration of with spatial displacement events not a power law, but rather presents an exponential cutoff, it is evidently affected by the temporal scale of observation with important consequences. Since 1 s sampling interval is very close to the exponential cutoff, overestimation occurs and caution is needed when extrapolations are considered.

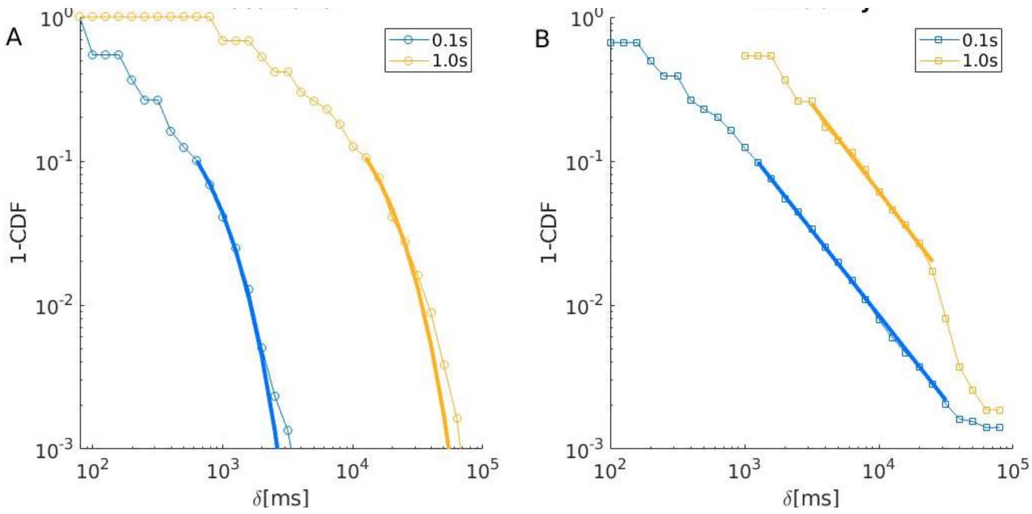

Supplementary Figure S2: Complementary cumulative functions of the same probability distributions shown in Figure 2 for both sampling rates. A) With displacement events show exponential cutoff. At higher exponential sampling the cutoff shift to the left. B) Without spatial displacement events show a power law distribution with exponent $b = 1$. Note the enlargement of the tail at a higher sampling frequency. Mathematical expressions and developments for CD's are presented in Kembro et al, (2019; Supplementary Material[2]) and considerations are taken into account when applied to the biological time series explored in Flesia et al. (2022)[3] .Thick lines highlight fittings.

Supplementary Table S1. Definition of behavioral variables recorded in social groups using ANY-maze video tracking system[4].

VARIABLE	DEFINITION
Peck	When one bird raises its head and vigorously pecks the other bird's body.
Grabs	When a bird catches ("grabs") with their beak the neck or head region of the other bird.
Mount	While performing a grab, the bird approaches the other bird from behind, and places both feet on the dorsal surface of its torso, stepping over the other bird's tail.
Cloacal contact	During mounting, the bird lifts his tail and tilts his pelvis underneath the other bird, and briefly presses its cloaca against the other bird.
Threats	One bird raises its head and neck rapidly and moves forward and backward vigorously in the direction of the opponent without making physical contact.
Chase	A bird runs after another that is escaping.
Foraging	Pecking at the ground or actively moving litter with the beak.
Feeding	Pecking at food in the feeding trough.
Dust bathing	Vertical wing shakes in a lying position .

Supplementary Table S2. Main features of Probability and Relative distributions (PD and RD, respectively) that characterize behavioral data of female and male quail within their home-box social groups (Experiment 1).

Behavior	Maximum value of PD (ms)	Maximum value of RD (ms)	Number of events	Span (orders of magnitude)
Grabs*	300	1000	28	1.5
Mount*	200	200	232	1
Cloacal contact*	400	1000	120	2
Feeding	300	10000	44	2.5
Drinking	500	4000	7	1
Pecking	200	200	273	1
Foraging	3000	3000	132	2
Dust bathing	200 & 7000	7000	342	3
Chasing	700	800	59	1.5

The definition of each behavior is provided in Supplementary Table S1. The peaks of the distributions are taken as the higher value of the respective histogram's bars. Note that such a value has a different range in the abscissa due to logarithmic binning. The span was measured as the support ($\delta_{max} - \delta_{min}$) of the distributions considering a logarithmic scale. *Reproductive behaviors exclusively performed by males.

Supplementary Table S3. Main features of probability and relative distribution (PD and RD, respectively) observed using high-resolution male Japanese quail behavioral data within novel social groups (Experiment 2).

Behavior	Maximum value of PD (ms)	Maximum value of RD (ms)	Number of events	Span (orders of magnitude)
Grab	63.1	398.1*	874	2
Mount	63.1	63.1	678	2
C. Contact	199.5	158.4	16	1.5
Reproductive +	***63.1***	***63.1***	***18***	***2***
Exploring	63.1	398.1	125	2
Walking	63.1	398.1*	1928	2.5
Running	398.1	398.1*	46	1
With displacement +	***63.1***	***1259***	***17***	***2***
Shaking	63.1	398.1*	52	1
Standing vigilant	199.5	398.1*	534	3
Resting	125.9	501.2	30	1.5
Standing	63.1	398.1 *	1580	2.5
Without displacement +	***63.1***	***63.1***	***22***	***3***

White cells represent characterization of PD and RD shown in Figure 6.
+ represent the recreated behavioral definitions shown in Figure 7.
*Peaks at 398.1 have an uncertainty of 100ms. ǂAll other non-displacement type events.

Supplementary Table S4. The vector B, which defines the borders of logarithmically sampled durations, and their corresponding minimum (emin) and maximum (emax) exponents for each experiment.

experim #	emin	emax	B [ms]
1	1.9	5	[79.4 100 126 … 63095 79432 100000]
2	1.5	5	[31.6 39.8 50.12 ... 63095 79432 100000]
3	1	8	[10 12.6 15.8 … 63095734 79432823 100000000]

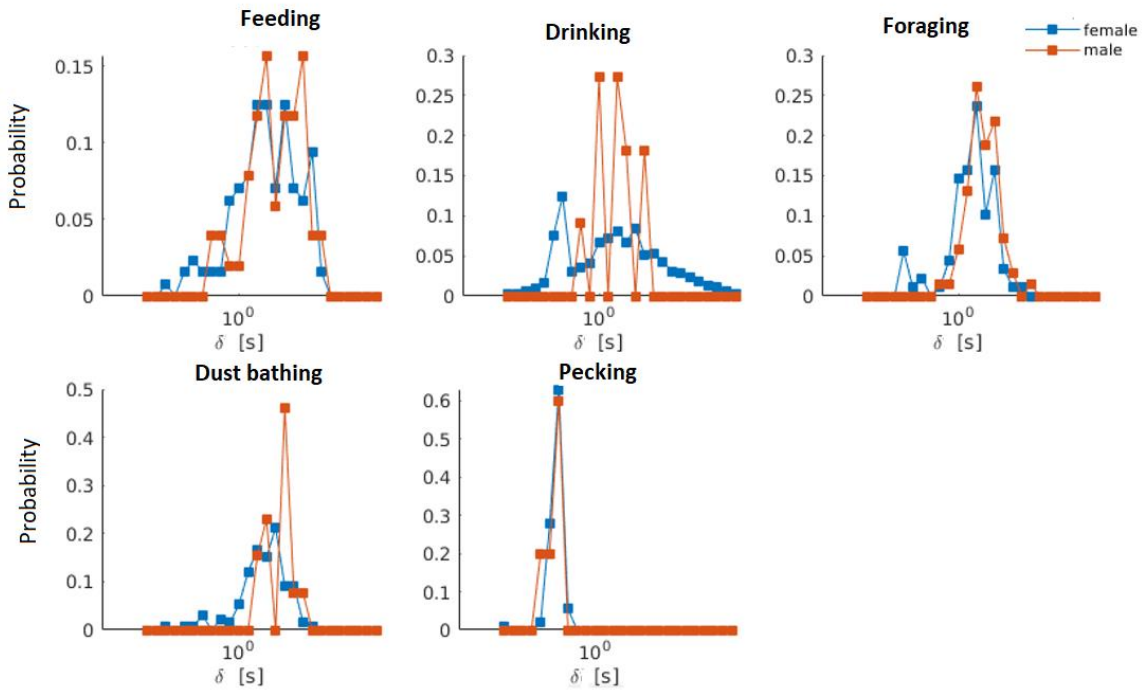

Supplementary Figure S3. Comparison between female (blue) and male (red) Japanese quail within social groups in regard to probability distribution functions of the duration (δ) of behavioral events.

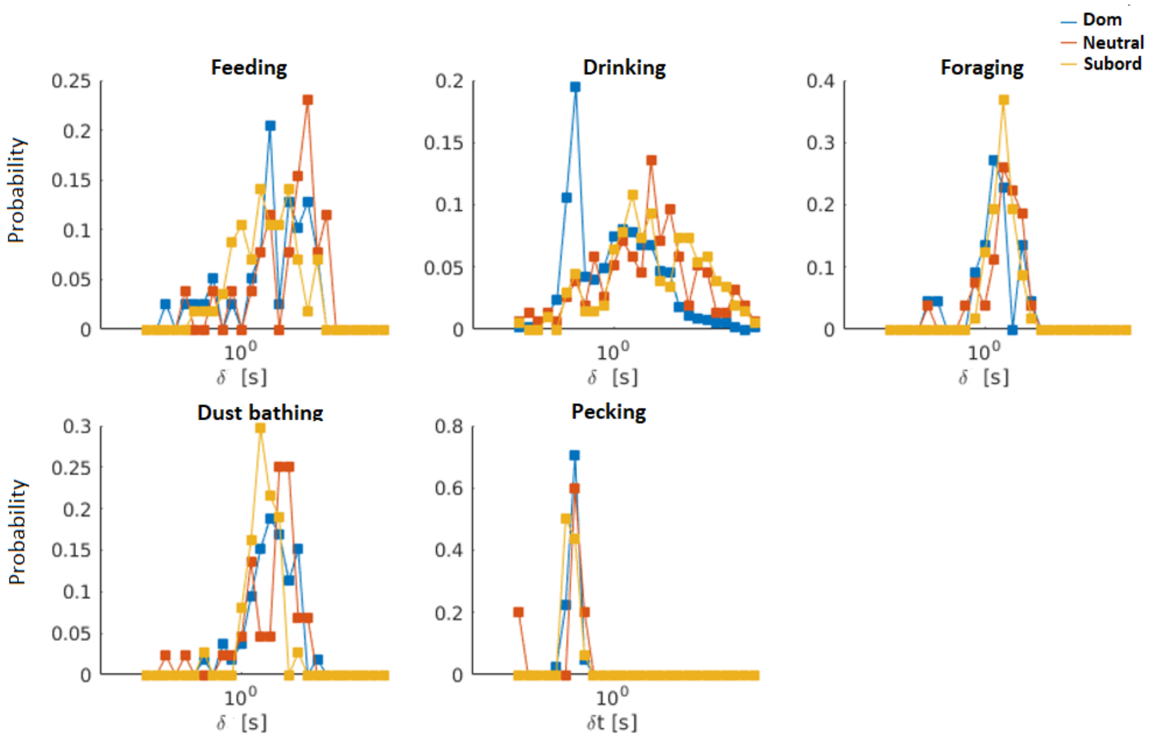

Supplementary Figure S4. Comparison between Japanese quail classified within social groups as dominant (blue), neutral (red) or subordinate (yellow) in regard to probability distribution functions of the duration (δ) of behavioral events.

References

1 Guzman, D. A. *et al.* The fractal organization of ultradian rhythms in avian behavior. *Sci Rep* **7**, 684, doi:10.1038/s41598-017-00743-2 (2017).

2 Kembro JM, Lihoreau M, Garriga J, Raposo EP, Bartumeus F. Bumblebees learn foraging routes through exploitation-exploration cycles. *J R Soc Interface* **16**, 20190103. doi: 10.1098/rsif.2019.0103. (2019)

3 Flesia AG, Nieto PS, Aon MA, Kembro JM. Computational Approaches and Tools as Applied to the Study of Rhythms and Chaos in Biology. *Methods Mol Biol* **2399**, 277-341. doi: 10.1007/978-1-0716-1831-8_13 (2022).

4 Caliva, J. M., Alcala, R. S., Guzman, D. A., Marin, R. H. & Kembro, J. M. High-resolution behavioral time series of Japanese quail within their social environment. *Sci Data* **6**, 300, doi:10.1038/s41597-019-0299-8 (2019).
